# Exposures and Health Risks from Volatile Organic Compounds in Communities Located near Oil and Gas Exploration and Production Activities in Colorado (U.S.A.)

**DOI:** 10.3390/ijerph15071500

**Published:** 2018-07-16

**Authors:** Tami S. McMullin, Alison M. Bamber, Daniel Bon, Daniel I. Vigil, Michael Van Dyke

**Affiliations:** Colorado Department of Public Health and Environment, 4300 Cherry Creek Drive S, Denver, CO 80246, USA; allie.bamber@state.co.us (A.M.B.); daniel.bon@state.co.us (D.B.); daniel.vigil@state.co.us (D.I.V.); mike.vandyke@state.co.us (M.V.D.)

**Keywords:** fracking, unconventional oil and gas, volatile organic compounds, VOC, hydraulic fracturing, air pollutants, health risk

## Abstract

The study objective was to use a preliminary risk based framework to evaluate the sufficiency of existing air data to answer an important public health question in Colorado: Do volatile organic compounds (VOCs) emitted into the air from oil and gas (OG) operations result in exposures to Coloradoans living at or greater than current state setback distances (500 feet) from OG operations at levels that may be harmful to their health? We identified 56 VOCs emitted from OG operations in Colorado and compiled 47 existing air monitoring datasets that measured these VOCs in 34 locations across OG regions. From these data, we estimated acute and chronic exposures and compared these exposures to health guideline levels using maximum and mean air concentrations. Acute and chronic non-cancer hazard quotients were below one for all individual VOCs. Hazard indices combining exposures for all VOCs were slightly above one. Lifetime excess cancer risk estimates for benzene were between 1.0 × 10^−5^–3.6 × 10^−5^ and ethylbenzene was 7.3 × 10^−6^. This evaluation identified a small sub-set of VOCs, including benzene and *n*-nonane, which should be prioritized for additional exposure characterization in site-specific studies that collect comprehensive time-series measurements of community scale exposures to better assess community exposures.

## 1. Introduction

**Figure 1:**
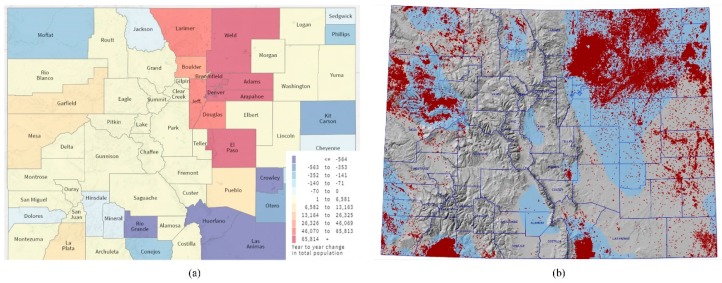
(**a**) Total population growth by county in Colorado (Data Source: Colorado State Demography Office, Department of Local Affairs (2018). Births, Deaths and Migration—Total Population Change 2008–2018. Retrieved from https://demography.dola.colorado.gov/ ComponentsOfChange) (**b**) oil and gas wells (red dots) in Colorado as of June, 2018 (Data Source: Colorado Oil and Gas Conservation Commission).

It is estimated that approximately 10% of Colorado residents live within one mile of an active oil and/or gas (OG) well [1]. Colorado currently ranks as the 5th state in natural gas production (1.7 million cubic feet in 2016) and 7th in crude oil production (9.1 million barrels in 2016) [2]. As of 2017, there were approximately 55,000 active OG producing wells, the majority unconventionally drilled wells. Over half of the wells are located within two counties, Weld (within the northern Front Range) and Garfield (on the Western Slope) [3]. Alongside OG growth, Colorado is also one of the top 10 fastest growing states in the U.S. [4]. Rapid population growth is occurring primarily in areas along the northern Front Range, on the surface of one of the largest OG basins in the U.S., the Denver-Julesburg basin (Figure 1). Current Colorado state regulations require that all new well or production facilities be located 500 feet or greater from building units (Colorado Oil and Gas Conservation Commission (COGCC Rule #604). However, prior to the COGCC rulemaking in 2013, setback distances were less than 500 feet from existing buildings. According to an analysis by McKenzie et al. (2016), approximately 0.8% and 0.1% of the people in the DJ and Piceance basins lived less than 500 feet from an existing OG well in 2012 [5].

Discussions regarding the many potential chemical and non-chemical health hazards affecting people living near OG operations have dominated the literature in recent years. In general, the hazards are similar to those that would be present in many industrial operations, including the potential for increased chemical exposures through air or water, physical hazards such as noise and vibration, safety hazards, and secondary hazards including psychosocial stress [6,7,8]. Of the chemical hazards, the process of OG extraction releases volatile organic compounds (VOCs) directly into the air. Multiple studies have identified several VOCs in the air near OG operations in Colorado [9,10,11,12,13,14,15,16,17,18,19,20,21,22,23]. Some of these VOCs are categorized as hazardous air pollutants by the U.S. EPA and Colorado Regulation 3 (5 CCR 1001-5) due to their potential to cause adverse health effects, such as cancer, neurological, developmental and reproductive effects and some have the capacity to alter endocrine activity at some exposure level [24].

In addition to hazard identification studies, a growing body of population based epidemiological studies have investigated associations between living near OG operations and the potential for certain health effects, such as adverse birth outcomes, upper and lower respiratory effects, cancer, and a variety of other health outcomes [25,26,27,28,29,30,31,32,33,34,35,36]. Although these epidemiological studies provide important insights into the potential health effects of at risk populations and assist in prioritizing public health research, one of the main limitations of these studies is the lack of direct quantification of chemical exposures to the observed population. In most of the epidemiological studies, proximity to an OG site has been used as a metric for exposures to the population of interest. This knowledge gap limits the utility of these studies in identifying and quantifying the specific chemical exposures that may be associated with the observed health outcomes.

**Figure 2:**
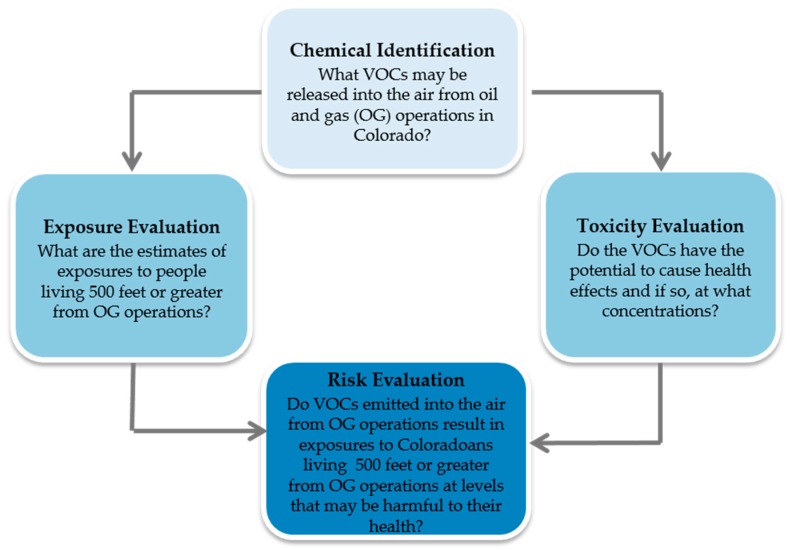
Four steps and associated questions that frame the scope of the screening level health risk evaluation.

Health risks from the VOCs emitted from OG operations are determined, in part, by the type and amount released into the air that could result in an exposure to someone living near these operations. There have been only a few studies quantifying the community level health risks of VOCs measured near OG operations or in OG regions in Colorado and other states. Some studies have been site specific while others were a broad evaluation of ambient air measurements [37,38,39,40,41,42]. Overall, the findings have shown mixed results. The generalizability of these studies to assess the potential for broad public health effects from VOCs to people living nearby these OG sites is limited, in part, due to varying factors, including small sample sizes, variability in geological formations, different operational processes across companies and regions, and significant changes in emissions regulations over time in Colorado. Recently, McKenzie et al. evaluated air monitoring datasets collected in the northern Front Range area of Colorado. The findings from McKenzie et al. suggested that health risks from air concentrations of VOCs increased as distance from an OG site decreased [37]. From a public health perspective, it is essential to evaluate all existing evidence, identify high priority VOCs that warrant further evaluation and identify key research areas where further data are needed to make sound, scientific risk management and public health decisions regarding health risks to people living outside current state setback distances from OG operations in Colorado. The objective of our study was to compile and conduct an exposure based screening level risk evaluation to determine the adequacy of existing ambient air data answer the following the question: Do VOCs emitted into the air from OG operations result in exposures to Coloradoans living at or greater than current state setback distances from OG operations at levels that may be harmful to their health? Four questions framed the scope of each step in the assessment (Figure 2).

## 2. Materials and Methods

Our methods used to conduct this preliminary risk assessment were consistent with U.S. EPA methods for assessing inhalation health risks using ambient air toxics data [43].

### 2.1. Chemical Identification

The first step in the assessment process was to evaluate existing data that could answer the question: What VOCs may be released into the air from OG operations in Colorado? This is an important first step in assessing the data to answer our key question regarding public health effects of VOCs directly emitted from OG operations, rather than assuming all VOCs identified in ambient air are directly emitted from OG operations. We found the following three sources of data that directly identified VOCs emitted into the air from OG operations in Colorado (Table S1).

- Operator emissions inventories submitted to the Air Pollution Control Division of the Colorado Department of Public Health and Environment (CDPHE), including gas and liquid analyses, emissions modeling and engineering calculations.
- Two studies that characterized VOCs directly emitted during different phases of O&G operations in the northern Front Range and Western Slope of Colorado [44,45].
- A source apportionment study that collected air data in OG areas of northeastern Colorado and modeled estimated percentages of OG source contributions to overall measured samples [13].

### 2.2. Exposure Evaluation

Upon identification of VOCs known to be released by one or several phases of OG development and production activities, we conducted a comprehensive review of existing air data across all OG regions of Colorado to attempt to answer the question: What are the estimates of exposures to OG related VOCs to people living 500 feet or greater from OG operations? As a screening level exposure assessment, we conservatively assumed that the outdoor air data represented exposure concentrations.

#### 2.2.1. Air Data Selection

**Figure 3:**
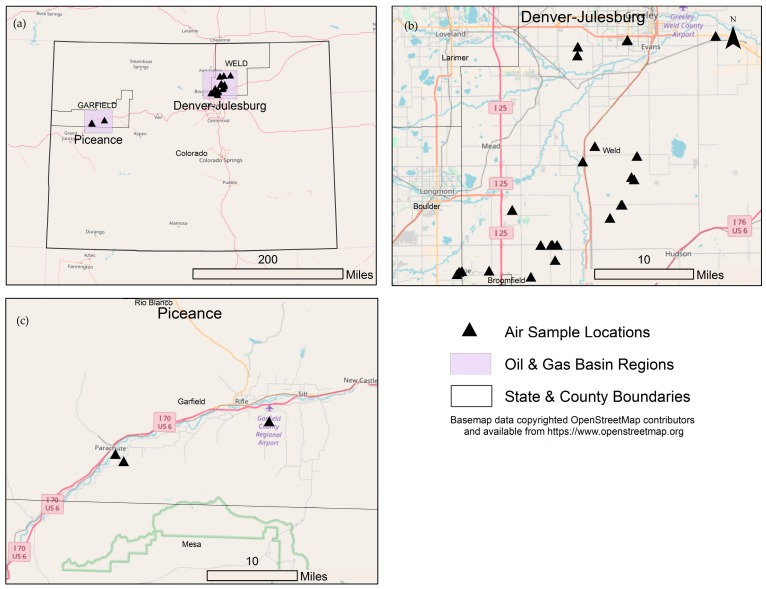
Air sample collection locations (**a**) within the two major oil and gas basins in Colorado, the (**b**) Denver-Julesburg Basin and (**c**) Piceance Basin. Table 1 provides details on individual air datasets.

We conducted a thorough search to locate ambient air data that measured the VOCs selected in Step 1 (Table 1). In order to ensure we evaluated the greatest number of samples possible, we used the following inclusion criteria:

- Original data from a study with clear objectives using standard air sampling analytical methods and analysis.
- Samples collected in a region of Colorado with substantial OG activity in the Denver-Julesburg and Piceance basins (Figure 3).
- Samples collected at heights that would generally represent ambient ground-level exposures. Aircraft measurements of VOCs were excluded. However, lower level stationary tower measurements (22 m) such as Swarthout et al. [19] were included.
- Samples that generally represented community scale exposures (middle to neighborhood spatial scales of representativeness as defined by U.S. EPA [46]) at 500 feet or greater from an OG site or multiple sites. The current setback distance is 500 feet from OG sites as established by Colorado Oil and Gas Conservation Commission (COGCC Rule #604). If sampling locations were specified by the study authors, we estimated the specific distance from an OG source by comparing distance from the air sampling location to the location of the nearest well pad using Google Earth and COGCC database for well locations [47]. One of the measurement sites in Thompson et al. [16] study was stated by the authors as being approximately 350 feet from an OG site while the other sites were 500 feet or greater. However, the study aggregated the data from all the sites and therefore, we conservatively chose to include the 350 foot sample site to allow inclusion of this dataset
- Samples collected during any type of OG operation, including processing, tank batteries, separators, pipelines, drilling, and production operations. Most studies did not identify the type of operation occurring during sample collection.
- Samples collected in an area that had minimal influence from other potential major sources of air pollution, including roads, industrial activities, or urban areas.
- In an effort to balance comprehensiveness and accuracy of our evaluation, we only used samples that were collected from 2008–2017. We did not use data collected prior to 2008 because significant regulatory changes occurred in Colorado that included major technological advances such as green completion technologies and data prior to 2008 would not be representative of current exposures (COGCC Rule 805.b).

Each site location was considered as an individual dataset. For sampling that was conducted at the same location for multiple years, each year was considered an individual dataset.

#### 2.2.2. Acute Exposure Estimates

For this screening evaluation, we define an acute exposure as a scenario in which a person breathes the outdoor air continuously for up to one hour at a time when concentrations of VOCs are highest. This assumed exposure duration is generally equal to the exposure duration used to derive toxicity values for comparison (Section 2.3). We selected the maximum VOC concentration across all studies as our dose metric for the acute risk characterization (Table S3). 

All datasets were included in the acute exposure assessment. We used the following process in the selection of maximum VOC concentration for each dataset:

- For time-series data that were collected at one hour or greater frequency intervals at a single location, we used the maximum measured air concentration for each VOC reported in the study [14,15,16,17,18,21].
- For time-series data that were collected at frequency intervals less than one hour and for which we could obtain the raw data from the authors, we generated one-hour averages and selected the maximum one-hour measurement [22].
- For data that were collected at frequency intervals less than one hour and but we could not obtain the raw data, we conservatively used the reported maximum measurement and assumed the value would represent a one-hour or greater air concentration [13,16,19].
- For one time air samples collected at a specific location, we assumed that the value could be a one hour maximum and included it in our analysis [20].

#### 2.2.3. Chronic Exposure Estimates

For this screening evaluation, we defined a chronic exposure as a scenario in which a person breathes the outdoor air continuously (24 h per day, 365 days per year) for a lifetime (i.e., 70 years) and the measured concentrations of the VOCs in the air remain constant over the entire lifetime [48,49]. Although drilling, hydraulic fracturing and flowback usually last days to several months and represent more sub-chronic rather than chronic exposures, we conservatively assumed all exposures could be chronic. Production may last 20–30 years and therefore, chronic exposures are possible.

Chronic HGVs generally represent continuous exposure greater than one year and up to a lifetime, depending on the agency. All datasets that represented a sampling duration greater than one single point in time were chosen for inclusion in the chronic assessment. For this reason, the single FRAPPE-WAS data samples were excluded [20]. To ensure that the highest average exposure of any type of operation and any distance was selected, we conservatively selected the highest mean VOC concentration across all the datasets and assumed this value represented a chronic exposure scenario for risk characterization. However, this could over-estimate risk because the highest VOC s used in the risk analysis could have come from different studies.

### 2.3. Toxicity Evaluation

The objective of the toxicity evaluation was to answer the question: Do the VOCs have the potential to cause health effects, and if so, at what concentrations?

#### 2.3.1. Non-Cancer

We used a tiered approach to select existing toxicity values from national and state sources in a manner consistent with U.S. EPA hierarchy guidelines [50] (Table 2). A higher tiered value was only selected if there was no value available from the lower tiers. For example, a Tier III value would only be used if there were no Tier I or II values available. For acute and chronic non-cancer health effects, state and federal agencies generally define these guideline levels as the concentration in the air below which health effects are not expected to occur, even for potentially sensitive people in the general population. For purposes of this evaluation, we call this level the health guideline value (HGV). This value is expressed in our paper as units of parts per billion. If needed, we converted values from µ/L assuming standard temperature and pressure. Ethane and propane do not have HGVs because of their lack of toxicity. We could not locate any agency established HGVs for four VOCs (ethylcyclohexane, 3-dimethylcyclohexane isomers and propylene (acute only)). Rather than evaluate them on a qualitative basis only, we used a surrogate approach based on evidence that these low carbon aromatics (C5–C8) would have similar toxicological effects as methylcyclohexane (Tier VI) [51].

#### 2.3.2. Cancer

We assessed VOCs as carcinogens if they were classified by International Agency for Research on Cancer (IARC) as a known (Class 1), probable (Class 2A), or possible (Class 2B) human carcinogen [52]. We selected agency derived inhalation unit risk (IUR) values according to our hierarchy (Table 2). The IUR is defined as the increase in the risk of an individual who is exposed for a lifetime to 1 µg/m^3^ carcinogen in air. These cancer risk estimates assume that any level of exposure to a carcinogen could pose a small, but finite, probability of generating a carcinogenic response. Benzene is classified by IARC as a known human carcinogen (1 Classification). We used the US EPA IUR range of 2.2 × 10^−6^ to 7.8 × 10^−6^ (µg/m^3^)^−1^. Ethylbenzene is classified by IARC as a possible human carcinogen (2B classification). Because US EPA has not derived an IUR for ethylbenzene, we used the CalEPA value of 2.5 × 10^−6^ (µg/m^3^)^−1^.

### 2.4. Risk Evaluation

#### 2.4.1. Non-Cancer Health Effects

We evaluated the potential for acute and chronic non-cancer adverse health effects using the following equations:

Acute HQ = Maximum air concentration/Acute HGV
(1)

Chronic HQ = Mean air concentration/Chronic HGV
(2)

HI = HQ_1_ + HQ_2_ + HQ_3_ (3)

where HQ is the hazard quotient, defined as the ratio of the exposure to the VOC to the level at which no adverse effects are expected (HGV). An HQ of less than or equal to 1 indicates that adverse non-cancer effects are not likely to occur, and thus can be considered to have negligible hazard, even for sensitive populations. An HQ greater than 1 does not translate to a probability that adverse health effects will occur and the magnitude of the HQ should not be interpreted to be proportional to risk. However, VOC exposures increasingly greater than the HGV (i.e., HQ greater than 1 suggests that the potential for adverse effects increases and the VOC exposures should be further evaluated.

#### 2.4.2. Carcinogenic Health Effects

To estimate excess lifetime cancer risks, we multiplied the highest mean exposure concentration across all datasets by the IUR value (or range of values for benzene). This represents the estimated increased excess cancer risk that a person would have if continuously exposed to the VOC concentrations for a lifetime [43,53].

#### 2.4.3. Health Effects from Combined Exposures

Since it is plausible that simultaneous exposures to multiple VOCs in the air can occur, we estimated the potential for non-cancer health risk from combined exposures using the hazard index (HI) approach. We used the U.S. EPA recommended dose-addition method in which all VOCs are assumed to cause health effects by a common mode of action or at the same target organ [48,54,55]. For purposes of an initial assessment, we combined all VOCs together. An HI of less than or equal to 1 indicates that adverse non-cancer effects are not likely to occur. VOC exposures increasingly greater than an HI of 1 suggests that the potential for adverse effects increases and further refinements to estimate the HIs for a subset of chemicals that have a similar mode of action or target organ should be evaluated along with additional analyses of the data that drive the HI greater than 1 [43].

## 3. Results

We identified 56 VOCs likely to be released into the air during some phase of OG operations in Colorado (Table 3; Table S1). Approximately 80% were located in Collet et al. [44,45], 55% were present in Gilman et al. [13], and 15% were in our operator emissions inventory (Table S1). Following identification of the VOCs emitted from OG operations in Colorado, we conducted a thorough search to locate all air monitoring data of the identified VOCs that met our inclusion criteria as a surrogate for exposures to people living 500 feet or greater from at least one OG operation. We found over 30,000 single air sample measurements across 47 different sets of air data collected across 34 locations (Table 1, Figure 2, Table S2). The actual study measurements were collected between 350–3700 feet from at least one OG site. Study durations and frequencies ranged from instantaneous passive whole air samples collected one time [20], one-second real-time measurements collected every minute for approximately one month [22], three-day integrated passive sampling every 6–10 days for approximately three months [15] to 24-h integrated canister samples collected weekly year-round for eight years [18]. Together, the data reflect seasonal and daily variability in VOC concentrations. Over one-third of the data were collected during nighttime and early morning when VOC concentrations often peak due to day-night atmospheric inversion cycles [13,14,16,19,21,22].

About 90 percent of the VOCs had chronic toxicity HGVs from federal (40%) or state (50%) agency databases (Table 3). Propane and ethane are simple asphyxiates with no systemic toxicity and therefore, had no HGV. We could not locate acute or chronic HGVs for only five VOCs (dimethylcyclohexane isomers, ethylcyclohexane, and propylene). We did not assess acute risks for propylene. For dimethylcyclohexane isomers and ethylcyclohexane, we conducted read-across using available scientific information on structurally similar analogues with established HGVs to fill toxicological data gaps (see methods).

**Figure 4:**
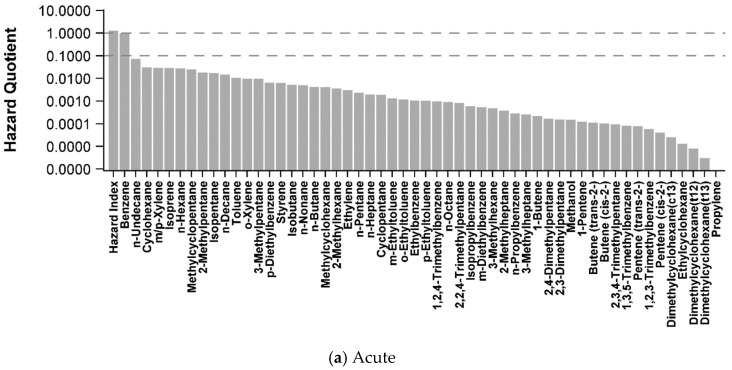

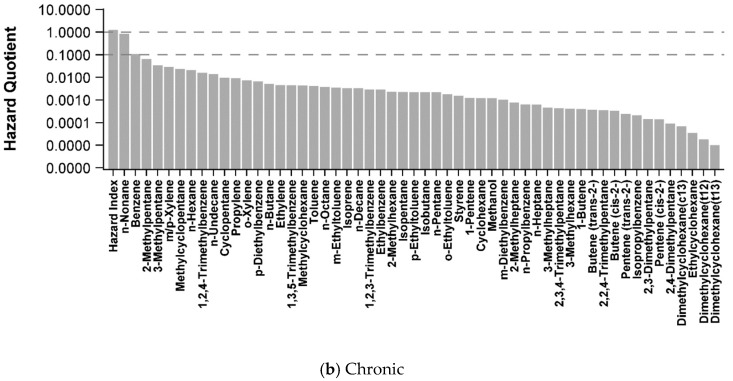
(**a**) Acute and (**b**) chronic hazard quotients (HQ) and hazard indices (HI) for 54 VOCs identified as emitted from OG operations in Colorado. The dotted lines indicate a HQ of 0.1 and 1. Note the log scale.

All air concentrations of individual VOCs were below acute and chronic non-cancer HGVs (HQ < 1) (Table 3, Figure 4). Of all 56 VOCs, benzene and *n*-nonane exceeded a chronic HQ of 0.1. The maximum level of benzene across all studies (8.6 ppb) approaches the acute HGV (9 ppb). Approximately 20% of the VOCs were about 60–100 times lower than their respective acute or chronic HGVs (HQ between 0.06–0.01). All other VOCs were 100–10,000 times lower (<0.01 in Table 3) (Figure 4). Combined hazard estimates for all VOCs were slightly above one for acute (HI = 1.2) and chronic (HI = 1.3) exposures (Figure 4). Only two of the VOCs are classified as known (benzene) and possible (ethylbenzene) carcinogens by IARC. Lifetime excess cancer risk estimates for benzene ranged between 1.0 × 10^−5^–3.6 × 10^−5^ and ethylbenzene was 7.3 × 10^−6^ (Table 4).

## 4. Discussion

We used a screening level risk based approach to evaluate the ability of existing data to answer the public health question: Do VOCs emitted into the outdoor air from OG operations result in exposures to Coloradoan’s living 500 feet or greater from these sites at levels that may be harmful to their health?

**Figure 5:**
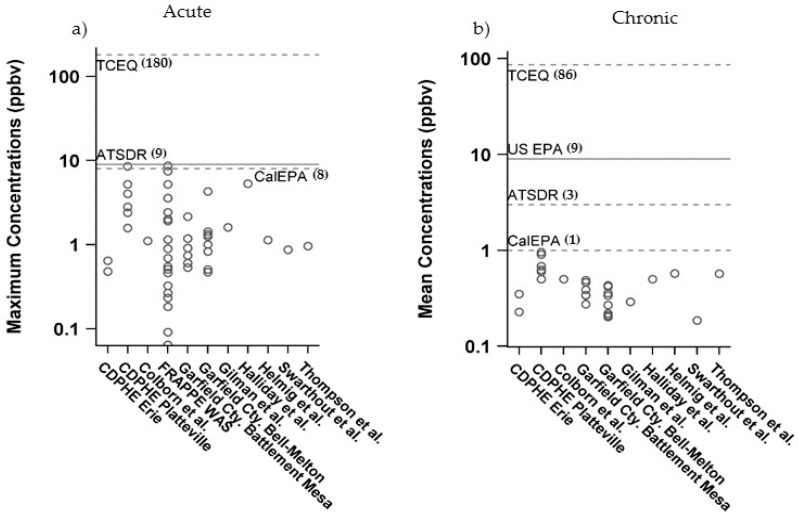
Comparison of distribution of (**a**) maximums and (**b**) means of air concentrations of benzene across all air data sampling studies to various agency derived (**a**) acute and (**b**) chronic health guideline levels (HGVs). See Table 1 for details of each study. Solid line represents the value chosen for this assessment. Brackets indicate the agency derived HGV.

One of our main goals in conducting this screening based risk evaluation of a broad set of ambient air data was to assist in identifying VOCs of greatest potential public health concern that may warrant additional study. Overall, we found that all individual VOCs were below levels that may pose non-cancer health risks and lifetime excess cancer risk estimates were around four in 100,000 or lower for inhalation exposures to people living at or greater than the current state setback distances (500 feet or greater from a OG site) in Colorado. Of all VOCs, benzene and *n*-nonane were the only VOCs with air measurements within 10-fold or less of their acute and/or chronic HGV’s (Figure 4). Our findings align with previous risk assessments that have identified benzene as the main VOC of potential public health concern. However, there is substantial inter- and intra-study variability and uncertainty in exposure and hazard data for benzene that is important to characterize further as part of evaluating the weight of evidence for health risks (Figure 5). As a first-tier screening level assessment, we conservatively chose the maximum measured air concentration for benzene as an estimate for acute exposures (Figure 5a) or the maximum mean benzene concentration for chronic, non-cancer exposures (Figure 5b) across all datasets and selected the federal agency derived HGVs for comparison, which was consistent with our selection criteria of preferentially using a federally established HGV. However, selection of a state derived HGV would have increased or decreased our hazard estimates. For example, acute HGVs for benzene vary by almost 200-fold between agencies, primarily due to differences in selection of exposure durations (i.e., 1 h vs. 14 day) and critical health endpoints used to derive the hazard values. These differences in HGVs result in a wide range of benzene non-cancer risk estimates for acute (0.05–1.1) and chronic (0.1–1) exposure scenarios, with CalEPA HGVs and one (chronic) or two (acute) air monitoring datasets driving HQ’s around 1 (Figure 5).

Combined acute and chronic HI’s to all VOCs were slightly greater than one. As a first screen, we conservatively chose to aggregate all VOCs to evaluate the potential for combined health effects to occur from exposure to the maximum (acute HI) or highest mean (chronic HI) air concentrations for all measured VOCs. There is a high level of uncertainty in the chronic HI for the following reasons: (1) The HI approach assumed all individual VOCs cause the same target organ health effects. However, over 70% of the total risk is driven by *n*-nonane (67%) and benzene (8.5%), which does not have similar target organ effects and no toxicological evidence that these compounds act by the same mode of action. The toxicological data used to derive the HGV for *n*-nonane is sparse and therefore EPA has rated their overall confidence in the value as low (2). The air concentration of *n*-nonane used to derive the chronic HQ for *n*-nonane is inconsistent with other datasets used in this evaluation. We used the highest average *n*-nonane measurement across all studies (3.3 ppb) from the Helmig et al. [15] study conducted in Eastern Boulder where the nearest OG activity was approximately 1500–2000 feet from the sampling site. However, all other studies collected at similar or closer distances directly in an OG region measured *n*-nonane at 0.2 ppb or lower. If the Helmig et al. [15] data were removed, the HQ for *n*-nonane would be 0.05 and the resulting HI would be 0.46. We have more confidence in the acute HI, where benzene drives over 75% of the acute HI. The maximum air concentration of benzene across all studies was from the CDPHE Platteville monitoring site (8.6 ppb). Two additional measurements in other studies were also over 8 ppb and approximately 60% of our air monitoring datasets had a maximum benzene level within 10 fold of its HGV. The main uncertainty in the acute HI assessment is the adequacy of the existing air monitoring data to represent acute exposures.

Although assessments from which to directly compare our risk findings are limited, conclusions from other studies that have evaluated community level health risks from OG operations have generally been consistent with our findings, particularly for chronic non-cancer and cancer health risks. A few site-specific screening assessments have been conducted in Colorado using a limited set of air data collected during specific activities occurring at an OG operations [21,37,41]. A similar approach to our study was previously conducted in Texas, in which the authors evaluated over 4 million ambient air measurements of VOCs to assess public health impacts from OG operations in the Barnett Shale region [38,39]. Both studies concluded that VOC air concentrations were consistently below federal and state acute and chronic HGVs. An additional study of potential health risks related to OG was recently conducted in Northeastern British Columbia [40]. OG emission data were used to generate model estimated exposures to multiple communities that would be impacted by potential OG activity. The modeling results from this study concluded that the potential for acute and chronic cancer and non-cancer health effects to occur in these communities from emissions from OG operations was low.

McKenzie et al. [37,42] conducted two assessments that can be most directly compared to our evaluation. McKenzie et al. [37], evaluated levels of VOCs in ambient air collected approximately 130 to 150 feet from the site during well completion at a site in Garfield County, CO when fluids from the well were put into open tanks venting directly to the air. Importantly, these samples were collected prior to new regulations requiring “green” or closed-loop completions. In general, the authors found that the VOC air concentrations closer to the well were elevated compared to the data collected further from the well. The authors concluded, therefore, that sub-chronic, but not chronic, hazard estimates for combined exposures to all VOCs were slightly greater than 1 for residents living less than ½ mile from a well. While the authors used standard screening level risk assessment methods and chronic HGVs consistent with our values, the results have limited comparability for those living at 500 feet or greater from the well primarily due to the assumption that the air concentrations measured at 150 feet from the OG site were indicative of exposures to people at distances up to ½ mile from a well.

More recently, McKenzie et al. [42], conducted a risk assessment using a sub-set of the data we used in this evaluation but also included measured VOCs within 500 feet from an OG operation to estimate exposures to people living at four categorical distances from an OG operation, within 152 m (within 500 feet), 152–610 m (500–2000 feet), 610–1600 m (2000–5249 feet), and >1600 m (>5249 feet) [42]. Similar to their previous assessment in Garfield County, the authors found that VOC concentrations increased with increasing proximity to an OG facility. Correspondingly, the acute and chronic HI’s and lifetime excess cancer risks for combined exposures for ethylbenzene and benzene exceeded one in 10,000 for exposures at distances within 500 feet from at least one OG facility. For this assessment, McKenzie et al. [42] primarily used CalEPA HGVs, which are slightly more conservative than the federal levels set by U.S. EPA (Figure 5). Nonetheless, their findings at distances greater than 500 feet were consistent with our results indicating individual VOC concentrations did not exceed acute and chronic non-cancer HGVs. In our study, the excess cancer risk estimate for combined exposures was 4.3 × 10^−5^ for distances between 500 feet and greater, which falls in the range reported by McKenzie et al. [42] for these same distances (5.7 × 10^−5^–1 × 10^−4^). Part of the air data used by McKenzie et al. [42] to assess acute and chronic non-cancer and excess cancer risks were measurements from ground based whole air samples collected in the FRAPPE campaign that provided a snapshot of VOC concentrations nearby multiple types of OG operations [20]. However, the measurements were either single 1-min whole air grab samples collected within 150 feet to a specified type of OG facility, or samples that were greater than 500 feet from a facility. Although the data within 150 feet may provide a more direct estimate of the VOCs directly emitted from specific types of operations, these air concentrations are not indicative of exposures at further distances from the sample location because VOCs demonstrate strong concentrations gradients over short distances and a high level of near-field variability [44,45,48]. Additionally, McKenzie et al. [42] extrapolated these one-minute samples to obtain a time weighted average over 24 h and then used these measurements to assess chronic exposure scenarios. We used these data to conservatively assume that these ground measurement FRAPPE samples collected at 500 feet or greater could be estimates of acute exposures. However, single one minute samples are generally not adequate duration for integrating over long time frames to estimate chronic exposures [43,48,49].

One of our initial study objectives was to identify a sub-set of VOCs measured in standard ambient air quality studies that are most likely emitted during activities associated with OG development and production processes in Colorado. There were over 150 compounds measured in one or more of the 47 air monitoring studies used in this assessment. However, we only identified 56 compounds directly emitted from OG operations. Although several published reviews have hypothesized that there are several hundred VOCs potentially emitted from OG operations, our evaluation of the data corroborates with other findings that there are a sub-set of VOCs measured in standard air monitoring protocols in which OG operations may be a main source contribution. A recent review of existing air measurement studies, modeling studies and qualitative evaluations of possible air pollutants in multiple states identified benzene, ethylbenzene, toluene, and styrene as VOCs most frequently identified across all studies [56]. Bunch et al. [39] evaluated inventory data reported by operators in Texas and identified 11 VOCs reasonably believed to be associated with shale gas operations in Texas, including benzene, ethylbenzene, xylenes (*m-, p-, o*-), *n*-hexane, and toluene. McKenzie et al. [37,42], identified a similar sub-set set of VOCs to our study with the exception of 1,3-butadiene (2012 study only). Although it is plausible that some OG activities, such as truck traffic and engine exhaust contribute to ambient measurements of 1,3-butadiene, this VOC was not identified in any of our primary sources of data as being emitted from OG emissions and was detected at a low frequency (~10%) in all ambient air samples (data not shown). Future studies that further characterize other primary and secondary pollutants related to OG emissions during different phases of operations, such as aldehydes, are needed to address the community level health effects.

The comparison of our study findings with previous risk assessments highlights the need for further discussions around the appropriate use of ambient air monitoring data to inform health risk assessments. The use of ambient air concentrations to estimate acute and chronic exposures is a standard way to screen a large set of monitoring data to identify substances that may pose the greatest concern and warrant further evaluation. The quality of the exposure estimate and consequent health risk estimate, however, depends on the methods used in the monitoring studies to derive these air concentrations. We used multiple air monitoring studies that measured VOCs over several years that met our inclusion criteria for use in a screening evaluation of community level exposures from OG operations. Overall, sampling collection methods, including sample numbers, timing (i.e., time of day and season), sampling durations and frequencies were highly variable across different studies primarily due to the different objectives of each air sampling campaign. Many studies were intended to assess the relative impact of OG activities on ambient air concentrations of VOCs and their relationship to ozone formation rather than for specific use in a risk assessment. Despite that limitation, many of the studies measured VOCs over a period of several hours to days on a weekly basis for several months to a year, which included seasonal and daily variations in air concentrations. These type of data are generally acceptable for generating average air concentrations that can be compared to chronic cancer and non-cancer toxicity values, which assume most conservatively, that outdoor air exposures are continuous over a lifetime [43,48,49]. We have less confidence that the existing ambient air data are of sufficient quantity and quality to represent maximum, intermittent acute exposure scenarios to potentially exposed populations of interest for comparison to acute toxicity values in a risk assessment. Three air monitoring studies in the DJ Basin collected samples at frequencies that ranged from one second to 5 min [13,19,22]. The site where short-term measurements were collected at the highest frequency was from the Halliday et al. [22] study, where one second samples were collected every minute for approximately one month at a location in the DJ Basin. Quantifying acute exposures is particularly challenging due to the extensive variability of VOC emissions from OG operations that leads to fluctuating types and concentrations of VOCs released during different phases of operation [57,58]. This variability is due to multiple dynamic factors, including local-scale meteorological conditions, operator specific processes, different durations of processes, and different geological formations. Future studies are greatly needed that focus on quantifying these acute, peak exposures to people living near OG operations, with particular emphasis on characterization of the VOCs identified as posing the greatest potential public health concerns, such as benzene. Ideally, future air monitoring data that are collected for use as exposure estimates coincide with the time frames used to derive toxicity values in order for the two types of data to be more appropriately combined for a risk assessment [48]. In addition, the samples need to be collected at frequent intervals in order to obtain the appropriate resolution for estimating acute health effects.

### Assumptions and Uncertainties

Consistent with a screening level risk evaluation of a broad set of diverse data, we used multiple conservative assumptions to minimize underestimation of exposure. Many of the main assumptions are stated in detail in the methods section. The following assumptions and uncertainties in this screening level risk evaluation could have resulted in an over or under-estimation the health risk and must be considered when drawing conclusions from the existing data:

- It is possible that the VOCs we selected as high priority for this risk assessment do not reflect the full profile of VOCs emitted from OG operations. Although we used several sources of information, including a study in which investigators quantified emission rates of VOCs directly from each phase of OG operations in Colorado, it is possible that there are other OG constituents or reaction products, such as higher molecular weight VOCs, aldehydes, ketones and alcohols that were not assessed in this study. Several additional VOCs were detected in the ambient air quality monitoring datasets but were not included in this initial screening assessment because there was no direct evidence that these VOCs were emitted from OG operations.
- We used ambient air data collected in regions with substantial OG operations as a surrogate for a person’s exposure, which could over or under-estimate risk. The data may not adequately represent community level exposures to people living at all distances between 500 and greater from oil and gas operations and therefore, may over- or under-estimate VOC exposures. In addition, the air data represent a person’s total outdoor air exposure to both OG and non-OG sources of emissions and therefore, may over-estimate the health risks solely from OG emissions. For example, several source apportionment studies in the DJ basin have indicated that traffic and non-combustion sources are a significant contribution to total ambient air measurements.
- We conservatively assumed that the air data represented a person’s exposure. A more refined exposure assessment that accounts for exposure scenarios that include locations of people in a study area and how those people move around during the day would likely decrease the risk estimates.
- We estimated combined health risks using the assumption of dose additivity. This assumes that all individual VOCs could act by the same mode of action and that interactions are not likely to occur at the exposure levels below their associated health guideline levels. Human health risk from exposures to chemical mixtures is complexly related to the pharmacokinetic and/or pharmacodynamic mechanisms of interactions that can lead to decreased or increased health effects. For example, there is some evidence that petroleum based mixtures containing benzene, toluene, ethylbenzene and xylene (BTEX) can lead to competitive metabolic inhibition at high concentrations and increase blood levels of BTEX that could result in increased health effects. However, multiple studies and toxicity assessments indicate that these interactions are negligible at environmental levels of exposures and therefore, the default assumption of additivity is sufficiently protective [59].

## 5. Conclusions

Based on our evaluation of existing air data collected in proximity to OG activities across Colorado, airborne VOCs are below levels that are anticipated to cause long-term non-cancer health effects to those living at distances of 500 feet or greater from these activities. Our analysis also suggests a low risk of harm from acute exposure to VOCs likely emitted from OG operations for residents at these distances, though this conclusion is tempered by the limited database of relevant air sampling data. Continued regional air quality monitoring along with more site-specific, community level air sampling is needed. As more comprehensive local-scale, community level or on-site air data are generated during different phases of OG operations, public health agencies will be able to develop a refined, limited set of VOCs with high potential to be emitted from OG operations. This information will allow agencies to optimize resources and conduct more targeted exposure characterization and risk assessment in potentially exposed communities. Specifically, air monitoring studies that continuously measure concentrations of a sub-set of high priority VOCs, during multiple phases of operations, and during times when citizens are experiencing health symptoms will provide critical exposure information necessary to refine current risk assessments. In addition, further characterization of primary and secondary VOCs emitted from OG sites during different phases of operations is needed to address the community health relevance of other potential VOC emissions not assessed in this study. Generating these exposure data is critical to developing scientifically sound risk management and public health policy decisions.

## Figures and Tables

**Table 1:** Summary of ambient air monitoring studies used for risk evaluation.

Reference	Description	Distance from Closest OG Site (ft)	Operation Type/Phase	Year(s) of Data Collection	Total Individual Samples Collected	Sample Collection Duration	Sampling Frequency and Duration	Time of Day	Season
*Denver-Julesburg Basin*									
CDPHE (Platteville) ^6^ [17]	air monitoring in oil and gas region with active development	950 ^4^	Various	2011–2017	369	3-h integrated	Weekly for 365 days	6–9 a.m.	All
CDPHE (Erie) [21]	site specific air monitoring	850–1650 ^3^	Completion	2012	36	3-h integrated	Every 3 days for 30 days then daily for 16 days	6–9 a.m.	Summer
FRAPPE WAS ^7^ [20]	site specific air monitoring	>500 ^4^	Various	2014	55	1-min grab sample	Once	Daytime	Summer
Gilman et al. ^1^ [13]	site specific air monitoring	984 ^3^	Production	2011	544	5-min integrated	Every 30 min for 19 days	24-h	Winter
Halliday et al. ^1^ [22]	air monitoring in oil and gas region with active development	1550 ^4^	Drilling, hydraulic fracturing	2014	28,009	1-s per minute	23 days	24-h	Summer
Helmig et al. [15]	air monitoring in region adjacent to active development	1470–2050 feet ^5^	Unknown ^5^	2014	47	3-day integrated	Every 6–10 days for 86 days	24-h	Summer
Swarthout et al. ^1^ [19]	air monitoring in oil and gas region with active development	984 ^3^	Production	2011	550	1-min grab sample ^2^	Hourly for 23 days	24-h	Spring
Thompson et al. ^1^ [16]	air monitoring in oil and gas region with active development	350–1400 ^3^	Production	2013	30	5-min grab samples, 3-h integrated, 24-h integrated	Random sampling over 77 days	24-h	Spring
*Piceance Basin*									
Colborn et al. ^1^ [14]	Site specific air monitoring	3700 ^3^	Drilling/production	2010/2011	48	24-h integrated	Weekly for 365 days	24-h	All
Garfield Cty. (Bell-Melton) [18]	air monitoring in oil and gas region with active development	500–800 ^3^	Various	2008–2015	390	24-h integrated	Weekly for 365 days	24-h	All
Garfield Cty. (Battlement Mesa) [18]	air monitoring in oil and gas region with active development	500–800 ^3^	Various	2010–2015	323	24-h integrated	Weekly for 365 days	24-h	All

^1^ Data are published in a peer-reviewed journal. ^2^ Data are from 22 m samples only. ^3^ Distance to nearest OG activity specified in study. ^4^ Distance to nearest OG activity was not specified in the study or there were multiple OG sites in the area. Approximate distances were based on study specified latitude/longitude air sample collection locations compared to the nearest active OG well using Google Earth and COGCC database for well locations. ^5^ No sample location or distance to nearest OG activity was specified in the study and distance was taken from McKenzie et al. [42]. The type of operation on the wellpad was not indicated. ^6^ Colorado Department of Public Health and Environment. ^7^ Front Range Air Pollution and Photochemistry Experiment—Whole Air Samples.

**Table 2:** Tiered approach for selecting acute and chronic non-cancer health guidance values (HGVs).

Tier	Source	Health Guidance Value
Tier I	U.S. EPA’s Integrated Risk Information System (IRIS)	Chronic: Reference Concentration (RfC) Cancer: Inhalation Unit Risk (IUR)
Tier II	Center for Disease Control—Agency For Toxic Substances and Disease Registry	Acute & Chronic: Minimal Risk Level (MRL)
Tier III	US EPA Peer-Reviewed Toxicity Values (PPRTV’s)	Chronic: PPRTV
Tier IV	California EPA (Cal EPA)	Acute and Chronic: Reference Exposure Level (REL) Cancer: Inhalation Unit Risk (IUR)
Tier V	Texas Commission on Environmental Quality (TCEQ)	Short & Long-Term: Air Monitoring Comparison Value (AMCV)
Tier VI	Surrogate approach	Not applicable

**Table 3:** Summary of combined air data, selected health guideline values (HGV) and calculated hazard quotients (HQs) for all non-cancer VOC’s. All concentration values in ppb; HQ are unitless.

Substance	Range of Mean Air Concentrations		Maximum Air Concentration	Acute HGV	Chronic HGV	Acute HQ	Chronic HQ
1,2,3-Trimethylbenzene	4.00 × 10^−3^	3.50 × 10^−2^	1.72 × 10^−1^	3000 ^5^	12 ^1^	<0.01	<0.01
1,2,4-Trimethylbenzene	1.80 × 10^−2^	1.90 × 10^−1^	2.90 × 10^0^	3000 ^5^	12 ^1^	<0.01	0.02
1,3,5-Trimethylbenzene	6.00 × 10^−3^	5.36 × 10^−2^	2.44 × 10^−1^	3000 ^5^	12 ^1^	<0.01	<0.01
1-Butene	1.29 × 10^−2^	9.12 × 10^−1^	5.92 × 10^0^	27,000 ^5^	2300 ^5^	<0.01	<0.01
1-Pentene	8.00 × 10^−3^	6.81 × 10^−1^	1.47 × 10^0^	12,000 ^5^	560 ^5^	<0.01	<0.01
2,2,4-Trimethylpentane	8.00 × 10^−3^	4.40 × 10^−2^	3.38 × 10^0^	4100 ^5^	124 ^3^	<0.01	<0.01
2,3,4-Trimethylpentane	8.00 × 10^−3^	5.30 × 10^−2^	3.84 × 10^−1^	4100 ^5^	124 ^3^	<0.01	<0.01
2,3-Dimethylpentane	3.10 × 10^−2^	3.15 × 10^−1^	1.26 × 10^0^	8200 ^5^	2200 ^5^	<0.01	<0.01
2,4-Dimethylpentane	2.40 × 10^−2^	2.00 × 10^−1^	1.34 × 10^0^	8200 ^5^	2200 ^5^	<0.01	<0.01
2-Methylheptane	3.40 × 10^−2^	3.00 × 10^−1^	1.54 × 10^0^	4100 ^5^	390 ^5^	<0.01	<0.01
2-Methylhexane	2.12 × 10^−1^	5.00 × 10^0^	2.94 × 10^1^	8200 ^5^	2200 ^5^	<0.01	<0.01
2-Methylpentane	3.60 × 10^−1^	3.69 × 10^0^	2.89 × 10^1^	1600 ^5^	57 ^5^	0.02	0.06
3-Methylheptane	2.40 × 10^−2^	1.79 × 10^−1^	1.04 × 10^0^	4100 ^5^	390 ^5^	<0.01	<0.01
3-Methylhexane	9.80 × 10^−2^	9.05 × 10^−1^	3.96 × 10^0^	8200 ^5^	2200 ^5^	<0.01	<0.01
3-Methylpentane	2.45 × 10^−1^	1.96 × 10^0^	1.52 × 10^1^	1600 ^5^	57 ^5^	<0.01	<0.01
Benzene	1.86 × 10^−1^	9.58 × 10^−1^	8.67 × 10^0^	9 ^2^	9 ^1^	0.95	0.11
Butene (cis-2-)	8.00 × 10^−3^	2.32 × 10^−1^	1.52 × 10^0^	15,000 ^5^	690 ^5^	<0.01	<0.01
Butene (trans-2-)	9.00 × 10^−3^	2.55 × 10^−1^	1.67 × 10^0^	15,000 ^5^	690 ^5^	<0.01	<0.01
Cyclohexane	1.43 × 10^−1^	2.09 × 10^0^	3.05 × 10^1^	1000 ^5^	1744 ^1^	0.03	<0.01
Cyclopentane	8.80 × 10^−2^	1.13 × 10^0^	2.02 × 10^1^	5900 ^5^	120 ^3^	<0.01	<0.01
Dimethylcyclohexane(cis-13-)	2.70 × 10^−2^	2.70 × 10^−2^	1.00 × 10^−1^	4000 ^6^	400 ^6^	<0.01	<0.01
Dimethylcyclohexane(trans-12-)	7.00 × 10^−3^	7.00 × 10^−3^	3.00 × 10^−2^	4000 ^6^	400 ^6^	<0.01	<0.01
Dimethylcyclohexane(trans-13-)	4.00 × 10^−3^	4.00 × 10^−3^	1.00 × 10^−2^	4000 ^6^	400 ^6^	<0.01	<0.01
Ethane	12.2 × 10^0^	1.39 × 10^2^	1.06 × 10^3^	NA	NA	NA	NA
Ethylbenzene	1.50 × 10^−2^	6.70 × 10^−1^	2.09 × 10^1^	20,000 ^5^	230 ^1^	<0.01	<0.01
Ethylcyclohexane	1.40 × 10^−2^	1.40 × 10^−2^	5.00 × 10^−2^	4000 ^6^	400 ^6^	<0.01	<0.01
Ethylene	4.34 × 10^−1^	1.12 × 10^1^	7.50 × 10^1^	25,000 ^5^	2500 ^5^	<0.01	<0.01
Isobutane	2.10 × 10^0^	2.19 × 10^1^	1.72 × 10^2^	33,000 ^5^	10,000 ^5^	<0.01	<0.01
Isopentane	1.60 × 10^−2^	1.80 × 10^1^	1.39 × 10^2^	8100 ^3^	8000 ^5^	0.02	<0.01
Isoprene	4.00 × 10^−3^	1.38 × 10^−1^	1.36 × 10^0^	48 ^2^	42 ^2^	0.03	<0.01
Isopropylbenzene	2.00 × 10^−3^	1.70 × 10^−2^	3.00 × 10^−1^	510 ^5^	81 ^1^	<0.01	<0.01
m-Diethylbenzene	4.00 × 10^−3^	4.70 × 10^−2^	2.38 × 10^−1^	450 ^5^	46 ^5^	<0.01	<0.01
Methanol	4.66 × 10^0^	1.83 × 10^1^	4.10 × 10^1^	270,000 ^1^	15,300 ^1^	<0.01	<0.01
Methylcyclohexane	1.43 × 10^−1^	1.74 × 10^0^	1.63 × 10^1^	4000 ^5^	400 ^5^	<0.01	<0.01
Methylcyclopentane	2.63 × 10^−1^	1.78 × 10^0^	1.83 × 10^1^	750 ^5^	75 ^5^	0.02	0.02
m-Ethyltoluene	1.00 × 10^−2^	8.65 × 10^−2^	3.31 × 10^−1^	250 ^5^	25 ^5^	<0.01	<0.01
m/p-Xylene	7.40 × 10^−2^	6.57 × 10^−1^	4.99 × 10^1^	1700 ^5^	23 ^1^	0.03	0.03
n-Butane	2.22 × 10^0^	5.17 × 10^1^	3.88 × 10^2^	92,000 ^5^	10,000 ^5^	<0.01	<0.01
n-Decane	1.00 × 10^−2^	5.75 × 10^−1^	2.58 × 10^1^	1750 ^5^	175 ^5^	0.02	<0.01
n-Heptane	1.34 × 10^−1^	1.38 × 10^0^	1.58 × 10^1^	8200 ^5^	2200 ^5^	<0.01	<0.01
n-Hexane	5.07 × 10^−1^	4.12 × 10^0^	4.46 × 10^1^	1600 ^5^	199 ^1^	0.03	0.02
n-Nonane	1.90 × 10^−2^	3.25 × 10^0^	1.49 × 10^1^	3000 ^5^	3.8 ^3^	<0.01	0.84
n-Octane	5.20 × 10^−2^	4.67 × 10^−1^	3.73 × 10^0^	4100 ^5^	124 ^3^	<0.01	<0.01
n-Pentane	1.05 × 10^0^	1.75 × 10^1^	1.60 × 10^2^	68,000 ^5^	8000 ^5^	<0.01	<0.01
n-Propylbenzene	4.00 × 10^−3^	3.24 × 10^−2^	1.44 × 10^−1^	510 ^5^	51 ^5^	<0.01	<0.01
n-Undecane	1.30 × 10^−2^	7.67 × 10^−1^	3.98 × 10^1^	550	55	0.07	<0.01
o-Ethyltoluene	3.00 × 10^−3^	4.49 × 10^−2^	2.92 × 10^−1^	250 ^5^	25 ^5^	<0.01	<0.01
o-Xylene	2.30 × 10^−2^	1.68 × 10^−1^	1.65 × 10 ^1^	1700 ^5^	23 ^1^	<0.01	<0.01
p-Diethylbenzene	8.00 × 10^−3^	3.00 × 10^−1^	2.90 × 10^0^	450 ^5^	46 ^5^	<0.01	<0.01
Pentene (cis-2-)	7.00 × 10^−3^	7.80 × 10^−2^	4.88 × 10^−1^	12,000 ^5^	560 ^5^	<0.01	<0.01
Pentene (trans-2-)	8.00 × 10^−3^	1.34 × 10^−1^	9.34 × 10^−1^	12,000 ^5^	560 ^5^	<0.01	<0.01
p-Ethyltoluene	5.00 × 10^−3^	5.55 × 10^−2^	2.56 × 10^−1^	250 ^5^	25 ^5^	<0.01	<0.01
Propane	5.21 × 10^0^	1.05 × 10^2^	7.23 × 10^2^	NA	NA	NA	NA
Propylene	1.04 × 10^−1^	1.61 × 10^1^	5.46 × 10^1^	NL ^7^	1744 ^4^	NA	<0.01
Styrene	5.00 × 10^−3^	3.63 × 10^−1^	3.09 × 10^0^	500 ^2^	235 ^1^	<0.01	<0.01
Toluene	1.90 × 10^−1^	5.49 × 10^0^	2.10 × 10^1^	2000 ^2^	1328 ^1^	0.01	<0.01

^1^ USEPA IRIS, ^2^ ATSDR, ^3^ US EPA PPRTV, ^4^ CalEPA, ^5^ TCEQ, ^6^ methylcyclohexane used as a surrogate, NA = not applicable, substance is a simple asphyxiate. ^7^ NL= not located in the literature.

**Table 4:** Cancer inhalation unit risk (IUR) values and excess cancer risk estimates for VOCs with carcinogenic potential.

Substance	Highest Mean Concentration (µg/m^3^)	IUR (Source)	Excess Cancer Risk
Benzene	4.6	2.2 × 10^−6^–7.8 × 10^−6^ (U.S. EPA) ^1^	1.0 × 10^−5^–3.6 × 10^−5^
Ethylbenzene	2.9	2.5 × 10^−6^ (CalEPA)	7.3 × 10^−6^
Aggregate Risk			4.3 × 10^−5^

^1^ Represents U.S. EPA’s lower and upper bound range of the IURs.

## Supplementary Materials

The following are available online at http://www.mdpi.com/1660-4601/15/7/1500/s1, Table S1: Selected VOCs for risk evaluation using source apportionment studies, Table S2: Details of ambient air monitoring studies used for risk evaluation, Table S3: Maximum and mean VOC concentrations from studies used in the risk evaluation.
